# Web‐based support services to help prevent suicide in young people and students: A mixed‐methods, user‐informed review of characteristics and effective elements

**DOI:** 10.1111/hsc.13819

**Published:** 2022-05-06

**Authors:** Rachel Cohen, Raphael Rifkin‐Zybutz, Paul Moran, Lucy Biddle

**Affiliations:** ^1^ Centre for Academic Mental Health Bristol University Medical School Bristol UK; ^2^ University Hospitals Bristol and Weston NHS Trust Bristol UK; ^3^ The National Institute for Health Research Applied Research Collaboration West (NIHR ARC West) at University Hospitals Bristol and Weston NHS Foundation Trust Bristol UK; ^4^ Population Health Sciences Bristol University Medical School Bristol UK

**Keywords:** mental health, students, suicide prevention, web‐based services, young people

## Abstract

The online world may provide an alternative means to engage young people and students with suicidal feelings, who are typically reluctant to seek help. We aimed to map, characterise and obtain user evaluation of current online suicide support for this group in order to assess the usefulness of current provision and how it may be improved. We conducted a mixed‐methods study, comprised of an internet search, content analysis of site features and qualitative interviews with site users: 9 young people and 4 general practitioners. Data collection took place in 2019 and 2020 in the UK. Young people participants were recruited through the well‐being networks of a large University in South‐West England and via a national young person's mental health app. General practitioners were recruited locally through professional networks. We identified a wide range of easily accessible online support, including examples of interactive services, such as live chat and text messaging, but a lack of support that is both suicide‐specific and young adult‐specific, and an absence of online suicide or mental health crisis support services tailored specifically for students. Qualitative data showed that clarity, brevity and immediacy are the most important facets of engaging crisis help for young people, and that young people may prefer to use text‐based rather than verbal forms of communication when seeking help. Few services provided access to active peer support, outside of lived‐experience stories, which were evaluated as both valuable and potentially harmful. There is a need to further develop tailored suicide specific online crisis support for young people and students, which is able to ‘speak to’ their age‐specific needs and preferences. While lived experience may provide a valuable means of supporting young audiences, caution is required since this may have unintended negative consequences and further research is needed to understand the safe framing of such material.

**What is known about this topic?**

- Young people are often reluctant to seek help for suicidal feelings from face‐to‐face services.
- Online mental health services do not always meet the needs or preferences of users in crisis.

**What this paper adds?**

- There is a lack of suicide‐specific online support for young people and students tailored to their age‐specific needs and preferences.
- When engaging with online services, young people seek immediate navigation to interactive support options and brevity in provision of psychoeducational content.
- Participants expressed a preference for text‐based live digital help offerings.

## 1 INTRODUCTION

Suicide is a leading cause of death amongst young people (YP) worldwide (Bilsen, 2018), but responding to this problem is challenging because YP are often reluctant to seek help (Michelmore & Hindley, 2012). The mental health of university of students has also been highlighted as a growing issue of concern in the UK, with rates of suicide increasing amongst this group (Gunnell et al., 2020) alongside reported low levels of help‐seeking (Knipe et al., 2018).

It has been suggested that the online world may provide an alternative means of delivering support (Robinson et al., 2017; Rowe et al., 2018; Seward & Harris, 2016). Use of the internet for purposes relating to thoughts of suicide is common amongst YP experiencing suicidal feelings (Mars et al., 2015) and may include seeking help, information or contact with like‐minded peers. An increasing range of suicide prevention material is available online (Thornton et al., 2017) yet seeking help online may also present risks (Marchant et al., 2017). For instance, Biddle et al. (2018) found online activity can maintain or exacerbate suicidal feelings since, even where initially seeking help online, users typically ‘stumble’ upon graphic content such as information about methods and display a tendency to flit between prevention and pro‐suicide content as mood lowers. Similarly, online interactions may espouse reinforcing attitudes towards self‐harm.

Adding to such difficulties, online services do not always meet the needs of users in crisis (Biddle et al., 2020). Evidence indicates that service features considered most helpful include immediacy of response in situ, and the provision of lived‐experience stories of self‐harm and interactive content, such as live chat, while signposting to external services (especially when requiring the user to make additional phone call or contacts) is less acceptable (Biddle et al., 2020). However, no studies have reported how commonly these features are provided by existing online services and few have explored users' perceptions of online help. We aimed to address these knowledge gaps via a comprehensive mapping exercise and in‐depth interviews with service users in order to provide a basis for improving online service provision for YP experiencing suicidal crisis.

## 2 MATERIAL AND METHODS

A sequential cross‐sectional mixed‐methods design was adopted. Ethical approval was obtained from the University of Bristol Faculty of Health Sciences Research Ethics Committee (ref: 92982). The study involved three phases:

### 2.1 Service mapping

We conducted an internet search to map existing suicide support provision for YP and students. We focused on services immediately accessible in a moment of crisis and therefore excluded Apps or social media requiring users to ‘sign up’ before accessing support.

The search comprised 18 search terms (Table 1) replicating a broad range of possible search strategies that might be used by YP and students specifically searching the internet for support during times of severe mental distress or suicidal feelings. The selected terms were informed by previous research (Biddle et al., 2018) and autocomplete search suggestions.

**TABLE 1:** Internet search core search terms

Suicide
Suicide help
Suicide help chat
Suicide helpline
Suicide helpline text
I want to die
I want to kill myself
I feel like killing myself
I'm too sad to live
I feel suicidal
Help me
Help me suicide
Feeling suicidal
Suicidal crisis
I'm having suicidal thoughts
Suicide safety plans
What are suicidal thoughts
I'm suicidal

**FIGURE 1:**
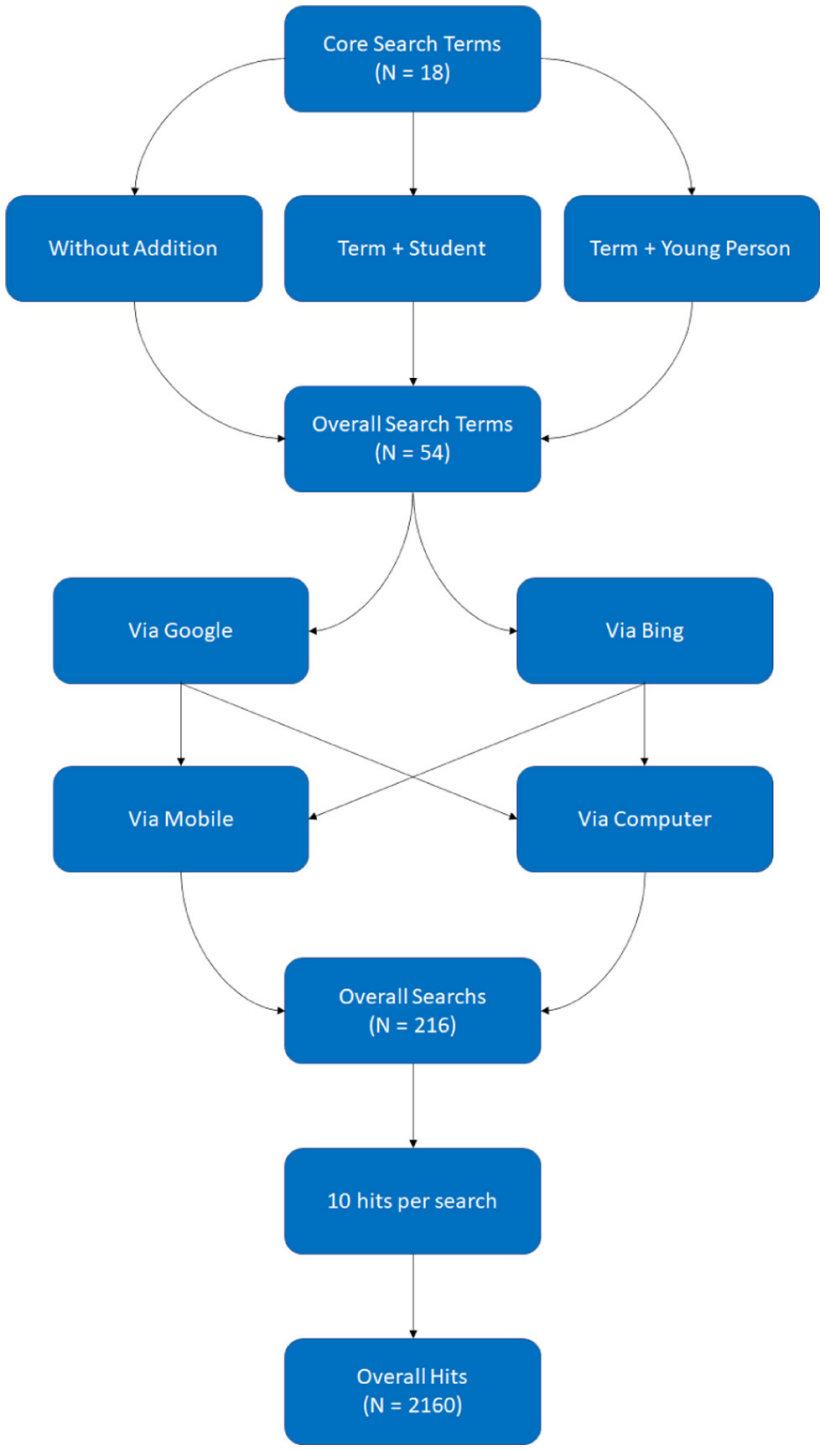
Search strategy

Terms were entered into the two most commonly used search engines in the UK in 2019 when the searches were conducted: Google and Bing, which jointly held 96.8% of the UK market share (www.statista.com). Searches were run on a desktop computer and mobile phone to capture disparities produced by searching on differing devices. To identify sites specifically targetting YP, each term was re‐run prefaced first with ‘young person’ and second with ‘student’, for example ‘suicide help’, ‘young person suicide help’, ‘student suicide help’. Each term was thus entered three times into each search engine and into both devices, resulting in a total of 216 searches. Consistent with other studies (Biddle et al., 2016), we retrieved the first 10 hits from each search (Figure 1).

Hits were classified by type using a coding frame. RC, RRZ and LB independently double‐coded then discussed a sub‐sample of hits. Existing coding categories were then refined, and new categories were created. This was repeated three times to establish a robust coding frame. RC and RRZ independently coded the remaining hits, meeting to discuss disagreements and refer uncertain cases to LB. A high inter‐rater agreement rate of 80% was achieved, with only 5% of disagreements requiring arbitration.

### 2.2 Content analysis

Hits coded as support/prevention services or as static support information pages were considered to be of most immediate relevance to the study aims and progressed to further content analysis (categories A and B, Table 2). Either a basic descriptive summary or a detailed analysis of website content was produced; detailed analysis being restricted to interactive services primarily dedicated to providing suicide and/or mental health prevention as these aligned most directly to our research aim. Detailed analysis, guided by a proforma, captured the key features of each service: types of support provided; resources (including downloadable materials); audience served (including diversity); strategies to communicate with users; and signposting to other resources. Analysis involved navigating away from landing pages to explore the whole site, including text, images and media.

**TABLE 2:** Frequency of hits by type

Code	Type with description	*n* (%)
A	*Support/prevention service*: offering a service beyond that contained on the webpage	57 (24.6)
B	*Static support pages*: offering information/advice or encouragement but no service	45 (19.4)
C	*Signposting information*: without accompanying support	16 (6.9)
D	*Advice/support for individuals supporting an individual with suicidal thoughts*	26 (11.2)
E	*User generated sites/pages*: such as chatrooms/forums	9 (3.9)
F	*Academic*/*policy pages*: presenting information about suicide in general or in young adults/students	24 (10.3)
G	*News reports*: discussing suicide in general or in young people/students	26 (11.2)
H	*Dedicated suicide sites*: entire sites dedicated to the discussion of suicide	1 (0.4)
I	*Suicide pages*: providing information or discourse about suicide which on occasion incorporates some help information, but this is not a focus. May include pro‐suicide discourse and/or encouragement, or ‘anti‐suicide’ discourse which ‘forbids’ suicide	28 (12.1)

### 2.3 Qualitative interviews with site users (YP and general practitioners)

In‐depth interviews were conducted with YPs and general practitioners (GPs) to gain evaluation of the format and content of online support services, and capture experiences of using, or referring to, such services. YPs were aged 16–25 years, English‐speaking and current/previous users of online crisis services. A convenience sampling approach was used to recruit participants by advertising through the wellbeing services of a large University in Southwest England and a UK‐based young person's mental health app: www.meetoo.help. We purposively sought students, recognising possible unique needs of this sub‐population relating to suicide prevention. GPs were English speaking and recruited via existing professional networks.

Interviews were semi‐structured and conducted via secure online video conferencing in 2020. Their length ranged from 22 min to just over an hour, with most lasting between 40 and 60 min. All participants were provided with a participant information sheet before commencing and gave informed consent. Interviews were audio recorded, then transcribed by a university‐approved transcriber in accordance with data protection regulations. Data collection and analysis were concurrent to ensure knowledge developed incrementally. A topic guide ensured our research aim was explored with each participant. This included broad headings and prompts relating to experiences of seeking online help during a crisis; the relationship between online and offline help; and evaluations of current online help content. Participants were also shown and asked to comment on two online service websites frequently retrieved in our search. However, participants were encouraged to talk freely identifying what they considered pertinent. The topic guide was revised to incorporate emerging themes.

Transcripts were coded by RC with a sample also independently coded by LB to ensure reliability. Coding involved identifying concepts and themes common across both participant groups and gathering supporting quotes. Content categories were developed within the themes through a process of independent analysis and then discussion between RC and LB. These captured the main considerations in relation to each theme were raised by YPs and GPs.

## 3 RESULTS

### 3.1 Service mapping

We retrieved 2160 hits in total. With duplicates, unavailable links and hits not relevant to suicide or mental health removed, 232 unique hits remained (Table 2).

Just over half of the unique hits (*n* = 118, 51%) were deemed to provide some explicit support for individuals experiencing mental health or suicidal crisis. Of these, the largest category (*n* = 57, 25%) was the online offering of larger support/prevention services—‘category A’. Thirty‐five ‘A category’ hits were provided by charity services (15% of all hits), 7 (3%) by statutory (NHS) health services, 13 (6%) by Universities whose services were available only to students at the particular institution, and 2 (1%) were private (psychiatry/CBT/counselling) services. The five most frequently retrieved hits (before removing duplicates) were all ‘A category’ sites. Other supportive hits were webpages providing static support information, reassurance or advice, but no contactable service beyond signposting to other organisations (‘category B’: *n* = 45, 19%); or pages comprised of signposting only, offering no significant in situ support (‘category C’: *n* = 16, 7%). A further small group of sites provided information for those supporting an individual with suicidal thoughts (‘category D’: *n* = 26, 11%).

The remaining 88 sites (40%) provided general information or commentary about suicide without specific focus on help or support, although reference to this could feature. These included academic/policy sites (*n* = 24, 10%), news media reports (*n* = 26, 11%) and user‐generated pages such as chatrooms or forums (*n* = 9, 4%). Only one such hit was an entire site dedicated to suicide and while not overtly pro‐suicide, substantial information was contained about suicide methods. This site appeared in 14 (7%) searches.

### 3.2 Content analysis of support/prevention services (‘A category’ hits)

Thirty‐five hits met the criteria for detailed content analysis—that is, they were deemed to constitute a *dedicated* support service and not merely static information. This was indicated by the site providing access to its own connected service components such as a phoneline or instant messaging. These were all ‘A category’ hits, linked to charitable services. One or more of these services appeared in 86.6% of searches. The majority were UK (*n* = 20) or USA (*n* = 11) based, with the remainder from Australia (*n* = 3) and Canada (*n* = 1). Content analysis of statutory, private and university services (also A category) was limited to basic descriptive summaries (reported below) since these were not wholly dedicated to providing support for mental health/suicide. Findings from detailed content analysis are summarised in Table 3.

**TABLE 3:** Key characteristics and help features of support/prevention service websites

	*N* (%) total *N* = 35
Site characteristics	
Focus	
Suicide only	11
Mental health, including suicide	24
Target audience	
Generic—Not‐specified	25
Young people specific	10
Student specific	0
Site includes content dedicated to specific minority groups (entire focus or dedicated pages)	10
Help features	
Help content provided at the site	
Suicide‐specific psychosocial education	21
Self‐help	19
Downloadable safety/crisis plan template	7
Peer support	4
Use of lived experience to deliver support messaging	17
Live digital interactive service hosted at the site	19
Text messaging	10
Online chat	12
Further help/support accessed via the site	
Access to own interactive services outside of the site, eg. Hotline	18
Signposting to other online services	28
Signposting to other offline services	29

#### 3.2.1 Site characteristics

##### Focus and target audience

We identified 11 suicide‐specific online help services. The remainder covered mental health more generally while referring to suicide. Most of the latter were pitched at a general audience, though 10 were YP specific. Notably, only 1 service was both young person *and* suicide specific (this appeared in less than half [40.7%] our searches), and no student‐specific suicide or mental health crisis services were found.

Most sites were deemed inclusive to a wide range of users, and some provided information for specific groups. Specialist LGBTQ support was provided by four sites, two sites provided gender‐sensitive information, one was tailored towards those of a Christian faith, and three provided support for deaf users. Some sites from the US/Australia offered support for First Nations people and Native Americans, and for disaster survivors. Images shown represented diversity; 17 sites included a range of ethnicities, 11 showed a range of different ages, and 10 showed images of different genders.

##### Help features provided at the service sites

The most common type of help provided was psychoeducation about mental health. Twenty‐one sites provided suicide‐specific information. Only one did not provide any such material, but instead emphasised the importance of reaching out for immediate help. Educational material typically incorporated a range of elements. Almost all sites highlighted the importance of seeking help. Over half offered advice and information on how to help someone else experiencing suicidal feelings. Approximately half strove to educate users about how to recognise warning signs associated with suicidality, and how to respond in a healthy way. Some included information on potential risk factors and possible causes of suicidal feelings. The presentational style varied but typically consisted of at least one page of material, mostly organised by sub‐headings and sometimes using bullet‐pointed text. The information presented by three sites was densely packed and detailed. Only two sites delivered information using videos aimed at someone who is suicidal.

Over half the sites (*n* = 19) offered tips or tools for self‐help. The scope of these ranged from self‐care around general mental health and wellness to more suicide‐specific advice; and from downloadable resources such as leaflets, factsheets and information on ‘how to cope’ (which were an extension to psychosocial education), to relaxation exercises and advice/tools for creating a suicide safety plan. Seven sites provided a downloadable crisis/safety plan template.

Half the sites (*n* = 17) included lived‐experience accounts of suicidal feelings as a means of communicating support information. Typically, these were framed as stories of ‘hope and recovery’ and several provided explicit accounts of people having successfully reached out for help, often emphasising the value of the support they had received from that service during a crisis. One site provided personal stories about coping with suicidal feelings more generally, and another provided accounts shared by two families bereaved by suicide. This content was presented in video format or on message boards or blogs. Lived‐experience was framed in collaborative terms: ‘helping each other out and going through similar stuff’ (www.childline.org.uk), or as a means of encouraging users to ‘seize the awkward’ (jedfoundation.org). Despite such emphasis, only four services enabled access to active peer support, this taking the form of a discussion forum or message board within the site. Two sites provided signposts to external peer support, such as online forums.

Each site was linked to its own interactive crisis service. For 19 (54%) services this consisted wholly or in part of a ‘live’ digital offering accessed at the site in the form of webchat or text messaging. All but three webchat services were available 24 h and 3 services provided both webchat and text messaging. While only 8% of the unique hits in our search provided such a facility, these hits emerged frequently with at least one appearing in 57.9% of our searches.

##### Further help accessible through service site

Twenty‐five sites provided their own interactive support(s) away from the site, such as an offline telephone hotline or email service. More than three‐quarters provided signposting to a range of further external online and offline support options. A similar cluster of online charitable services was commonly signposted (all included in our analysis). Offline services included 999 Emergency Services, local GPs and regional counselling services. Signposted support options were typically limited to the country/location of the originating site, so may not be accessible to international users.

#### 3.2.2 Basic descriptive summaries

Statutory pages provided brief, text‐based psychoeducational information and coping strategies for people experiencing suicidal thoughts. All signposted to a range of support services, and some included statutory emergency numbers and reference to GPs. None featured videos. Three focused on YP. University pages provided basic information about suicide and suicidal thoughts, but most primarily signposted to other support services, including the Universities' own offline wellbeing services. One university page featured lived experience videos and blog/vlog links. Private services (*n* = 2) were not suicide or young person‐specific and provided some psychoeducational content and minimal signposting. Static support sites (category B) were exclusively composed of psychosocial education and signposting. In all instances, charity services were chiefly signposted to as the main source of online suicide support, reaffirming the focus of our detailed content analysis on these services.

### 3.3 Qualitative findings

Nine YP (3 male, 6 female) and 4 GPs (3 female, 1 male) were interviewed after responding to advertisements for research participants. YP had a mean age of 19 years, two were university students and all but one had sought online help for mental health difficulties. Some had also sought offline healthcare, for instance from primary or secondary care. All shared their views on the likely value of online help in times of suicide or mental health crisis. GPs were experienced in providing mental healthcare to YP, all with over 10 years of experience, and two specialised in student mental health.

#### 3.3.1 Age‐appropriate support

All participants thought YP engage more effectively during a mental health crisis where site content is tailored using age‐appropriate language. One YP described how a negative experience in this respect led her to ‘*emotionally distance*’ [YP8] herself, while another noted it was ‘*quite nice to get advice from someone your own age*’ [YP4]. GPs observed YP are less likely to resist mental health support delivered in an age‐sensitive way.

#### 3.3.2 Psychoeducation

Young peoples and GPs argued strongly that online psychoeducation needs to be clear and brief—especially during mental crisis when large quantities of information were deemed overwhelming and likely to discourage a young person from staying with the site:

If someone is at their rock bottom and really distressed, they can barely process information and so they really need it to be written in a very simple and straightforward way. [GP2]

People just want … some bite size information with a quick introduction in order to decide whether it was for them or not or whether it was relevant. [GP4]

Underlining this, one YP praised the simplicity of the information provided by a service website examined during interview, surmising this would support their engagement and noting how this contrasted to other examples:

[named site] doesn't bombard you with a load of information, and it's kind of keeping it short and sweet, you know, it doesn't make you feel overcrowded … [it] summarises it down so you can kind of feel at peace with yourself. [YP9]

Ease of navigation was also important and layouts permitting the young person to filter their reading by quickly identifying relevant sections:

[named site] has clear headings, that really matters for me … sometimes, I just read the subheading and I don't want to read every single word like skimming through … [on named site] people can search for different topics immediately, like being more specific to their problem. [YP1]

#### 3.3.3 Lived‐experience content

Almost all YPs thought lived experience material about suicidal feelings is helpful as it can reassure that others confront similar difficulties and offer hopeful examples of successful recovery.

I think that gives like reassurance to someone who might be reading it and like, ‘the other people have felt this, people know how I'm feeling’. [YP3]

When you find someone that's been in a similar situation and you see them doing well and stuff like that, it gives you that hope you can be there 1 day. [YP7]

GPs acknowledged similar benefits

[It] helps with just knowing they're not alone; there are others who have been through similar things, especially success stories … Those messages of hope are quite important. [GP4]

YP also suggested stories may help YP recognise and validate crisis—often a pre‐requisite to help‐seeking:

It's good just to show suicide as being manifested differently … so if you're not feeling this one certain thing, that doesn't discount your feelings … it's good to show all the different things that might come under [feeling suicidal]. [YP2]

It's so useful because you might feel like sometimes you don't know whether you're experiencing [crisis] yourself, so if you hear someone else saying what they've been through you might relate to them and be like, ‘Oh yeah, I'm going through the same thing’. [YP4]

Further, lived experience was valued as an engaging way to deliver support information and prompt action:

This [part of site] is very directed at someone who would be in a mental health crisis … it says, ‘If you have thoughts about suicide like I do, please read this’ … Like someone else is going through this and it invites you to read it rather than just being like, ‘Oh, here's some suicide help.’… it's like ‘read my experience’ and then you can kind of relate to it and see what you need to do. [YP3]

However, it was acknowledged that such content also had potential to be harmful. GPs, in particular, expressed concerns that it could inadvertently lead YP to feel that they have ‘failed’ to recover at the same pace as the person portrayed:

[On some sites] you can watch about how people feel or how they've recovered …. Obviously, you have to be careful about the messages. For instance, if someone is feeling very desperate and they see that someone else has got better, they might feel more of a failure, but I think there's obviously a way to frame that. [GP2]

One YP cautioned that stories could be triggering, providing a contrasting perspective to those who thought they might help YP identify distress:

There can be stories about people who have had suicidal thoughts over a certain [issue] … I know my friends get really triggered by that kind of thing, so it's kind of a touchy subject. They're sometimes really useful, but sometimes they can do more damage. [YP9]

#### 3.3.4 Interactive crisis support

YPs highlighted immediacy is crucial for those seeking help during crisis, who need support urgently and may not have sufficient emotional resources to explore websites for necessary details. They praised sites which enabled users to navigate to support without delay, by making their help options (e.g. helpline numbers or online chat portals) readily identifiable and accessible.

There's a lot of articles on the front that you have to scroll through. And then you click the [links] and it gets you to the ‘Get support’ … it would probably be better if it was like right on the front page … so you can like click on that instead of having to like look through all the articles and get a bit confused about where to go. [YP3]

YP were clear such information should be prioritised over psychoeducation information:

For me … the first thing was, I need to talk to someone, I don't want to read about why I feel this way … The main thing is just having that as like the first thing you see is just call us if you need something. [YP6]

One YP emphasised that a desire for ‘immediate’ information and speedy solutions is characteristic of YP more generally, and that service providers would do well to acknowledge this to maximise the likelihood of engaging young users, whether presenting support options or psychosocial information:

[Named service] is good, it has like a number at the top … it feels like you know where to go directly … [young people] just want to get things resolved quickly. Like especially teenagers, they're in the period of time where they don't like reflecting, they want answers … even like having a list of bullet points, some people … might not even read them … they're not calm, they can't wait, they want to have it done, get it resolved quickly. [YP1]

YPs and GPs also argued that online crisis support should offer services in a range of different communicative formats to meet YP's often diverse communication preferences, though YPs expressed a preference for written communication—text, webchat—over verbal methods (e.g. phone) when reaching out for help during crisis. They found verbal interactions intimidating, particularly when experiencing high levels of anxiety, and found it easier (sometimes calming) to articulate difficult feelings in writing:

It can be quite hard to even get the words out when you're struggling. I've found that, you can't even like get the words out to explain what's wrong. Whereas like if you got a text, you've got the time to get across what you want to say. It can be less nerve wracking … than having a conversation. [YP8]

Texting things is a so much easier way of communication and it does bring your anxiety down … picking up the phone, ringing someone and having someone answer would make you freeze a bit … texting someone, you can get a massive, big, long message and break it down and so you really get out everything you feel. [YP5]

## 4 DISCUSSION

This study mapped online help provision for YP and students experiencing acute mental or suicidal crises. We identified a wide range of easily accessible online support, either focusing or touching upon suicidal thoughts or behaviours and ranging from signposting and static information to interactive services. Fifteen per cent of unique hits were prevention services, wholly dedicated to providing mental health or support for suicidal crises. These amounted to 35 charity services, of which at least one appeared in 87% of our searches, and four were amongst the five most frequently retrieved sites. Our findings show an individual entering basic suicide‐related help search terms (e.g. suicide help) also will commonly be directed to general discourse around suicide, which on occasion could trigger harmful behaviour in vulnerable individuals. This included one site containing detailed information about suicide methods, retrieved in 7% of searches.

Content analysis of dedicated interactive online crisis support services revealed that while there were several mental health services aimed at YP, only one was suicide specific, and none were dedicated to students as a specific population group. However, in qualitative interviews, YP and GPs stressed the importance of age‐appropriate support to meet users' needs and secure engagement. Provision of psychoeducational information was the most common service feature, offered by almost all services. Self‐care advice was also common with several services providing downloadable documents, such as safety plan templates. Qualitative findings indicated that YP preferred psychoeducational material to be filterable, brief and clear because lengthy information was described as overwhelming and lacked immediacy.

Interview participants deemed the most effective services as those whose interactive help options were displayed clearly and immediately—for example, on a running banner or on the website homepage—so they could be accessed as the first line of response without having to read other information. It was highlighted that crisis support options should be provided in a range of formats, though there was a strong preference amongst participants in this study for written (e.g. webchat and text), rather than verbal (e.g. phoneline), forms of communication when seeking help online. Nineteen of the services provided online chat and/or text messaging, and one of these services was reached in 57.9% of our searches.

Over half the services utilised lived‐experience accounts as a means of engaging users and delivering support messaging. While the YP interviewed were largely positive about this, perceiving it to offer reassurance, hope, interpretation of symptoms, and validation, a potential for unintended harm was also flagged. Only a few services offered access to active peer support.

It is asserted that the internet can contribute to suicide prevention (Luxton et al., 2012). Our study has provided the first comprehensive mapping and characterisation of current online provision of help for YP and students experiencing suicidal crises and has explored the prevalence of features suggested by other research (Biddle et al., 2020) as most likely to meet the needs of distressed users. While identifying examples of valuable online support, we found a lack of opportunities for peer support and support that is both suicide‐specific and young adult‐specific. This is notable since research indicates online users with suicidal thoughts have unique help needs that are not well met by general mental health sites (Biddle et al., 2020). Moreover, we found an absence of suicide or online mental health crisis support services tailored specifically for students despite growing concern about the mental health of YP in higher education (Reavley & Jorm, 2010). As well as aligning with current understanding about preferences for online help, our qualitative findings also provide additional insights into engaging ways of presenting information and making sites navigable and YP's preferences around the format of ‘live’ online help offerings. Participants' reflections on lived‐experience content are pertinent to existing debate about the benefits of such material (Till et al., 2017) and reinforce the need for research able to specify ingredients of safe and supportive lived experienced accounts.

### 4.1 Strengths and limitations

Our mixed‐methods design allowed us to quantify the features of online suicide crisis provision while simultaneously providing a more nuanced understanding of how these are experienced by users. Interview participants were recruited from diverse settings to increase the experiences represented. The YP drew on experiences of acute mental distress and online service use and their accounts were triangulated with those of GPs with expertise in adolescent and student mental health. This approach furthers previous work, which has sought participant views via survey methods (Bell et al., 2018; Harris et al., 2009). While our participants provided rich accounts with extensive (often longitudinal) narratives of seeking online support for mental distress and consistent themes were obtained within our analysis, our sample size was small and further research with purposive sampling would be beneficial to examine transferability of findings.

Our study was conducted in England and whilst not limited to UK‐specific services, this may have influenced our search output and we did not seek to incorporate international perspectives about online service provision. The online world constantly evolves and responds to the changing social context. Our mapping provides a cross‐sectional view and some changes to online provision are likely to have occurred over time in the same way that accessibility of pro‐suicide content has also been observed to change (Biddle et al., 2016). For instance, upon briefly revisiting some of the sites identified, we observed provision of additional interactive crisis support options during the COVID‐19 pandemic: one of these was student specific. We also noted changes to the images on several sites since the Black Lives Matter protests of 2019, and that a wider range of ethnicities was now depicted. Although none of the sites provided dedicated support for ethnic minority groups at the time of our mapping exercise, several UK services now incorporate a greater number of signposts to specific sources of support for these groups. Despite the fluidity of online provision, our findings highlight generalisable observations about which service features and styles are considered effective and engaging for YP in need of crisis support: knowledge which can provide useful guidance for future service development. They also identify aspects of service provision that require further research. In particular, while lived experience may provide a valuable means of engaging and supporting young audiences, participants flag that caution is required since this may have unintended negative consequences. Further research is needed to understand variations in interpretation and safe framing of such material.

Due to our focus on immediately accessible crisis services, our mapping did not include mental health apps available to YP. The support provided through apps may be more age‐specific and likely to include features such as peer support, which was limited in the online settings studied here. Further research is necessary to map such support and understand characteristics of users and patterns and experiences of app engagement in times of crisis. The functioning of online peer support also requires further investigation, including where risks may arise and how these can be managed.

While providing a comprehensive mapping of available sites and their features based on likely search terms, our study cannot replicate the actual online navigation of distressed YP nor account for online features such as personalisation of news feeds or targeted advertising based on search history. We cannot therefore comment with accuracy on the frequency at which particular sites are accessed.

## 5 CONCLUSIONS

Our study identifies a need to develop online crisis support that is both young person and suicide‐specific and provides essential information to aid the design of such services. Our qualitative data highlight clarity, brevity and immediacy as the most important facets of engaging crisis help for YP. This should include information and advice specifically tailored towards this age group since YP may be discouraged from engaging with services that do not ‘speak to’ their age‐specific needs and preferences, or where they feel that their difficulties are not validated by the forms of support available. To address YP's desire for quick responses, ease of navigation should be improved by placing crisis support options upfront and making these immediately visible while ensuring brevity in the provision of psychoeducational content. Providing more opportunities for active peer support may also help to address the desire expressed by YP to interact with others having similar experiences, and online crisis services could provide the ideal forum for ensuring this is offered in a moderated environment. The growth of live digital help offerings such as instant messaging and texting is a welcome development, which speaks to our participants' preferences for written rather than verbal forms of crisis help and might usefully be expanded, though the needs of users with low literacy should also be remembered. Such a service was reached in only just over half (57.9%) of our searches, indicating that a potentially valuable opportunity for more effective suicide prevention is still being missed.

## CONFLICT OF INTEREST

None declared.

## AUTHOR CONTRIBUTIONS

LB and RC conceived of and designed the study. RC and RRZ ran internet searches and carried out the content analysis with support from LB. RRZ conducted quantitative analysis. RC collected the qualitative data and led analysis with support from LB. All authors participated in study development meetings. LB and RC prepared the manuscript. RRZ and PM commented on manuscript drafts. All authors read and agreed the final manuscript.

## Data Availability

The data that support the findings of this study are available from the corresponding author upon reasonable request.
